# Decrease in active hepatitis C infection among people who use drugs in Madrid, Spain, 2017 to 2023: a retrospective study

**DOI:** 10.2807/1560-7917.ES.2024.29.29.2300712

**Published:** 2024-07-18

**Authors:** Pablo Ryan, Jorge Valencia, Guillermo Cuevas, Rafael Amigot-Sanchez, Isidoro Martínez, Jeffrey V Lazarus, Felipe Pérez-García, Salvador Resino

**Affiliations:** 1 Hospital Universitario Infanta Leonor, Madrid, Spain; 2 Universidad Complutense de Madrid (UCM), Madrid, Spain; 3 Instituto de Investigación Sanitaria Gregorio Marañón (IiSGM), Madrid, Spain; 4 Centro de Investigación Biomédica en Red en Enfermedades Infecciosas (CIBERINFEC), Instituto de Salud Carlos III, Madrid, Spain; 5 Unidad de Reducción de Daños ‘SMASD’, Madrid, Spain; 6 Unidad de Infección e Viral e Inmunidad, Centro Nacional de Microbiología, Instituto de Salud Carlos III, Majadahonda, Madrid, Spain; 7 Barcelona Institute for Global Health (ISGlobal), Hospital Clínic, University of Barcelona, Barcelona, Spain; 8 CUNY Graduate School of Public Health and Health Policy (CUNY SPH), New York, United States; 9 Servicio de Microbiología Clínica, Hospital Universitario Príncipe de Asturias, Madrid, Spain; 10 Universidad de Alcalá, Facultad de Medicina, Departamento de Biomedicina y Biotecnología, Madrid, Spain

**Keywords:** Hepatitis C, mobile screening unit, people who use drugs, people who inject drugs, Spain, epidemiology

## Abstract

**Background:**

People who use drugs (PWUD) are a key target population to reduce the burden of hepatitis C virus (HCV) infection.

**Aim:**

To assess risk factors and temporal trends of active HCV infection in PWUD in Madrid, Spain.

**Methods:**

We conducted a retrospective study between 2017 and 2023, including 2,264 PWUD visiting a mobile screening unit. Data about epidemiology, substance use and sexual risk behaviour were obtained through a 92-item questionnaire. HCV was detected by antibody test, followed by RNA test. The primary outcome variable was active HCV infection prevalence, calculated considering all individuals who underwent RNA testing and analysed by logistic regression adjusted by the main risk factors.

**Results:**

Of all participants, 685 tested positive for anti-HCV antibodies, and 605 underwent RNA testing; 314 had active HCV infection, and 218 initiated treatment. People who inject drugs (PWID) were identified as the main risk group. The active HCV infection rate showed a significant downward trend between 2017 and 2023 in the entire study population (23.4% to 6.0%), among PWID (41.0% to 15.0%) and PWUD without injecting drug use (7.0% to 1.3%) (p < 0.001 for all). These downward trends were confirmed by adjusted logistic regression for the entire study population (adjusted odds ratio (aOR): 0.78), PWID (aOR: 0.78), and PWUD non-IDU (aOR: 0.78).

**Conclusions:**

Our study demonstrates a significant reduction in active HCV infection prevalence among PWUD, particularly in PWID, which suggests that efforts in the prevention and treatment of HCV in Madrid, Spain, have had an impact on the control of HCV infection.

**Key public health message**

**What did you want to address in this study and why?**

There is a high prevalence of hepatitis C (HCV) among people who use drugs and consequently they are at risk of developing liver cirrhosis, which can progress to liver decompensation, hepatocellular carcinoma and death. We wanted to identify the main risk factors for HCV infection and understand how the prevalence has changed over time from 2017 to 2023. We used a mobile screening unit in Madrid, Spain, to study HCV prevalence in people who use drugs.

**What have we learnt from this study?**

Our findings confirm that injecting drug use is the most prominent risk factor for active HCV infection. The overall rate of active HCV infection among people who use drugs visiting our mobile screening unit notably decreased from 23.4% in 2017 to 6% in 2023, confirming the effectiveness of current prevention and treatment strategies. However, HCV infection remains high among those who inject drugs.

**What are the implications of your findings for public health?**

The reduction in HCV infection rates among people who use drugs suggests that HCV screening programmes targeting this at-risk population helps achieve the World Health Organization goal of HCV elimination by 2030. However, the persistently high rates of infection among injecting drug users highlight the need for interventions to reduce the burden of HCV infection in this high-risk group. Monitoring HCV prevalence trends continuously among high-risk groups is crucial.

## Introduction

The World Health Organization (WHO) estimates that around 50 million people worldwide live with hepatitis C in 2022 [1]. The hepatitis C virus (HCV) is transmitted by exposure to contaminated blood, causing chronic hepatitis C in around 70% of people with HCV infection. Hepatitis C virus infection promotes the development of liver fibrosis, cirrhosis, hepatic decompensation, hepatocellular carcinoma (HCC) and death over the years [2]. In 2020, the estimated viraemic HCV infections globally were around 0.7% [3]. In line with this, the latest seroprevalence survey in Spain (2017–18) showed an overall HCV seroprevalence of 0.85%, an active HCV infection prevalence of 0.22%, and an undiagnosed fraction of active HCV infection of 29.4% [4].

The introduction of direct-acting antivirals (DAAs) in the early 2010s marked a revolution in HCV infection treatment, achieving sustained virological responses in ≥ 95% of treated patients and with an excellent reported safety profile [5,6]. Since then, DAA therapy is recommended for all individuals with HCV infection [7], leading to a considerable increase in its use and a decrease in the incidence of hepatitis C [8]. Despite this, hepatitis C remains a major health problem because many people still face barriers to access to treatment. The WHO has set goals to reduce the prevalence of hepatitis C considerably by 2030 [9]. This initiative involves a scale-up of screening, risk behaviour reduction and unrestricted access to treatment of HCV infection. However, only around 36% of people infected with HCV globally were aware of their diagnosis in 2022, and about 20% were treated [2]. The burden of HCV infection among people who use drugs (PWUD) is higher than in the general population, even though the HCV prevalence has decreased in recent years [10]. This is particularly relevant in people who inject drugs (PWID) [11,12], where HCV transmission is frequent because of ongoing risk behaviours [13,14]. The most common risk factor for HCV infection in high-income countries is injecting drug use (IDU), which is responsible for ca 70% of new HCV infections [5]. In Spain, before the introduction of DAAs in PWID, the prevalence of active hepatitis C in PWID was ca 40–60% [15,16].

The WHO strategy also highlights the importance of targeting key populations to reduce the burden of HCV-related diseases, such as PWID [9]. In line with this, evaluating trends in HCV prevalence and risk factors for active HCV infection is crucial for micro-elimination strategies [17]. Although there is evidence to support an optimistic view that Spain is on the right path towards HCV micro-elimination among PWID [18,19], other reports indicate that risk behaviour among PWID fuels new HCV infections and reinfections [20], being a key obstacle in Spain to reach the WHO goal regarding HCV.

In the Spanish public health system, all people have the right to receive treatment against HCV infection. Nevertheless, vulnerable populations face barriers to accessing healthcare when using traditional models of care, such as primary care centres. Therefore, harm-reduction services provide frequent blood-borne infection testing and support to PWUD to guarantee access to hepatitis C care and treatment [14]. Micro-elimination of hepatitis C in PWID depends on periodic HCV screening, extensive treatment and the sustainability of harm-reduction strategies to prevent future HCV infections [21]. These interventions, including the provision of opioid substitution therapy (OST) and needle and syringe programmes, have proven to reduce both the incidence of HCV primary infection and reinfection among PWID [22]. Additionally, HCV treatment as prevention strategies [23] and harm-reduction interventions are vital for reducing or eliminating HCV in PWID and other key populations [23].

Universal HCV screening is recommended because it is cost-effective, crucial, and necessary for any HCV elimination strategy [5]. Previous works have shown that HCV screening through a two-step point of care (PoC)-based strategy using a mobile screening unit and its linkage to care was highly effective for identifying and treating marginalised people with active hepatitis C [16,18]. We aimed to assess the risk factors and temporal trends of HCV infection among PWUD at a single mobile screening unit from Madrid, Spain, between 2017 and 2023 in the DAA era.

## Methods

### Study setting and population

We conducted a retrospective study among active PWUD in Madrid, Spain, from 1 June 2017, to 3 April 2023. Participants were recruited consecutively in the order of appearance in Madrid's geographic ‘hotspots’. These hotspots are areas where high-risk individuals gather for dealing and drug consumption, and because of their characteristics and risk factors, they are more likely to have a higher prevalence of hepatitis C. Such locations include institutions providing social assistance, homeless shelters, mobile harm reduction units, public areas and places where street prostitution is practiced. Screening for HCV was offered to everyone. Inclusion criteria for this study were: (i) age ≥ 18 years and (ii) active drug use in the year before HCV screening.

We defined PWUD as those with drug consumption over the past year. We stratified PWID into two groups based on their injection activity within the past year: active or inactive. This stratification was determined by the time elapsed since their last injection.

### Hepatitis C virus screening

A mobile screening unit consisting of a van adapted for HCV screening and a support car approached the hotspots (n = 42 places) at least once per year and following a predefined schedule. A nurse collected a capillary blood sample for HCV screening. Between 2017 and 2018, these blood samples were collected using dried blood spots (DBS) on Whatman cards, which were sent to the Instituto de Salud Carlos III (ISCIII) for HCV diagnosis by ELISA (Murex anti-HCV kit, v. 4.0, DiaSorin) and in-house PCR for HCV infection (HCV-PCR), as previously described [16,24]. Between 2019 and 2023, capillary blood samples were analysed by the OraQuick HCV Rapid Antibody Test (OraSure Technologies) and an HCV-PCR test with the PoC Xpert-HCV-VL (Cepheid), as previously described [18]. Our general strategy was that those participants with a positive anti-HCV antibody result would undergo an HCV-PCR test.

Moreover, the screening mobile unit team investigators collected data about demographic, epidemiology, substance use, and sexual risk behaviour through a questionnaire on a mobile device with an internet connection, as previously described [16,18].

### Outcomes

The primary outcome was the presence of an active HCV infection in PWUD among those screened by a mobile unit. The secondary outcome was the initiation of HCV treatment among PWUD screened with active HCV infection.

### Statistical analysis

Statistical analyses were made with Stata IC 17 (StataCorp) and IBM SPSS v24 (IBM Corp,). GraphPad Prism v9.0 (GraphPad Software, Inc.) generated graphics. All p values were two-tailed, and p < 0.05 was considered statistically significant.

For descriptive analysis, categorical variables were shown as absolute count (percentage) and quantitative variables as median and interquartile range (IQR). The chi-square test was used to compare categorical variables. The Mann–Whitney U test was used to compare continuous variables. The active HCV infection rate was calculated for all individuals who underwent HCV-RNA testing. We evaluated risk factors (patient characteristics) for primary (active HCV infection) and secondary (initiation of HCV treatment) outcome variables by multivariate logistic regression, taking into account patient characteristics, providing the adjusted odds ratio (aOR), their 95% confidence intervals (CIs) and the area under the receiver operating characteristic curve (AUC). We also analysed the temporal trend of active HCV infection rates by calendar years using multivariate logistic regression adjusted by patient characteristics. Patient characteristics included in multivariate logistic regression were age over 50 years, sex (female/male), birth country (Spain, Eastern Europe, Western Europe, Africa, America and others), homelessness, benzodiazepine use, alcohol use, drug consumed (cocaine, heroin and cannabis), IDU, OST, other administration route (smoked and snorted) and sexual intercourse in the past year (never, condom use and no condom use).

## Results

**Figure 1:**
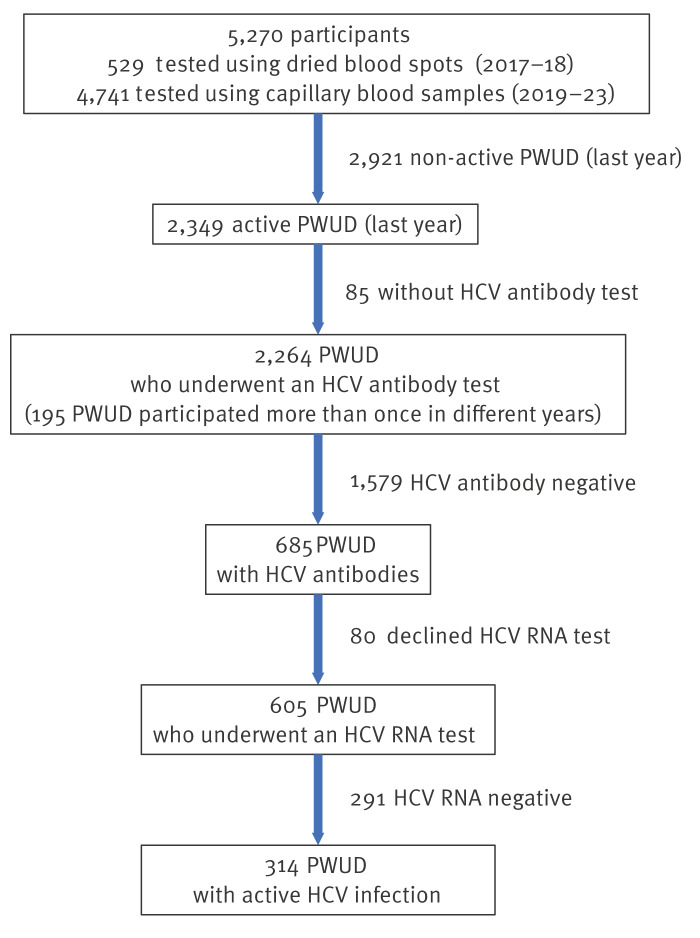
Flowchart of the study population of PWUD and results of the diagnostic tests, Madrid, Spain, 1 June 2017–3 April 2023

HCV: hepatitis C virus; PWUD: people who use drugs; RNA: ribonucleic acid.

Of 5,270 participants in the initial cohort for hepatitis C detection, 2,349 (44.6%) were active PWUD. Only 2,264 (43.0%) underwent an HCV antibody test, and 195 (8.6%) of them participated more than once in different years throughout the study period. In total, 2,525 antibody tests and 2,414 HCV-RNA tests were performed. Of those 2,264 PWUD, 685 (13.0%) tested positive for anti-HCV antibodies, and 80 of these 685 participants (11.7%) declined HCV-RNA testing and were excluded from the analysis. The main reason for not performing the HCV-RNA test was that they had already had a previous HCV-RNA test recently at another centre. Other reasons include the proactive outreach nature of the intervention targeting a highly transient population of active drug users, many of whom are often in a hurry, transient or reluctant to undergo testing. Additionally, some participants left immediately after the HCV-antibody test, and a few encountered a technical error with the HCV-RNA test and chose not to wait for a retest. Regarding the 605 participants who underwent HCV-RNA testing, we found 314 (51.9%) with active HCV infection (**Figure 1**), which showed a median HCV viral load value of 470,500 (IQR: 32,625–1,817,500) IU/mL. Finally, of 314 participants with active HCV infection, 292 (92.9%) received the test results, 240 (76.4%) voluntarily booked an appointment at the hospital, and 218 (69.4%) started HCV treatment.

### Participant characteristics

Table shows the epidemiological characteristics of the PWUD population. Overall, PWUD who underwent an HCV antibody test had a median age of 43 years; 76.6% (n = 1,735) were male, 27% (n = 612) were migrants with 9.8% (n = 222) from Eastern Europe, and 45.9% (n = 1,027) were homeless. Regarding substance use, 38.1% (n = 856) had a daily alcohol use (> 50 g/day), and 23.8% (n = 539) used benzodiazepines. Cocaine and heroin were the most frequently consumed illegal drugs (90.2% (n = 2,041) cocaine and 64.3% (n = 1,456) heroin), and 34.9% (n = 770) were under OST. Additionally, 43.4% (n = 983) were PWID, of whom 52.7% (n = 518) had recent IDU, and 72.4% (n = 1,640) PWUD had sexual intercourse in the past year (35.3% (n = 579) of them without condom use). Of the total, PWUD with active HCV infection were significantly older and had higher proportions of European origin (Eastern Europe and Western Europe), homelessness status, drug consumption (cocaine and heroin), IDU, and OST than PWUD without active HCV infection. By contrast, PWUD not infected with HCV had lower percentages of migrants of African and American origin, alcohol use, marijuana use and consumption of smoked and snorted drugs (Table).

**Table:** Epidemiological characteristics of PWUD according to active hepatitis C infection status, Madrid, Spain, 1 June 2017–3 April 2023 (n = 2,264)

Characteristics	HCV antibody test		HCV active infection				p value
	All		No		Yes^a^		
	n	%	n	%	n	%	
Number of participants	2,264	100	1,870	82.6	314	13.9	-
Female sex	529	23.4	446	23.9	62	19.7	0.111
Male sex	1,735	76.6	1,424	76.1	252	80.3	0.111
Median age in years, (IQR)	43 (35–50)		42 (34–49)		44 (38–50)		< 0.001
Age > 50 years	499	22.3	394	21.3	67	21.5	0.922
Source population							
Spain	1,652	73.0	1,350	72.2	232	73.9	0.534
Eastern Europe	222	9.8	159	8.5	57	18.2	< 0.001
Western Europe	60	2.7	42	2.2	15	4.8	0.009
Africa	145	6.4	142	7.6	3	1.0	< 0.001
America	133	5.9	129	6.9	4	1.3	< 0.001
Other	52	2.3	48	2.6	3	1.0	0.080
Stable residence							
Homelessness	1,027	45.9	821	44.4	162	52.1	0.012
Substance use							
Alcohol use^b^	856	38.1	719	38.7	95	30.5	0.006
Benzodiazepine use	539	23.8	442	23.6	72	22.9	0.785
Drug consumed							
Cocaine	2,041	90.2	1,668	89.2	295	93.9	0.010
Heroin	1,456	64.3	1,129	60.4	259	82.5	< 0.001
Marijuana	491	21.7	442	23.6	35	11.1	< 0.001
IDU							
History of IDU	983	43.4	647	34.6	267	85.0	< 0.001
Active (past year)	518	52.7	326	50.4	167	62.5	< 0.001
Opioid substitution therapy	770	34.9	564	30.9	156	50.5	< 0.001
Other administration routes							
Smoked	1,853	81.8	1,554	83.1	235	74.8	< 0.001
Snorted	508	22.4	475	25.4	29	9.2	< 0.001
Risky sexual behaviour							
Sexual intercourse (last year)	1,640	72.4	1,361	72.8	234	74.5	0.520
No condom use	579	35.3	464	34.1	94	40.2	0.072

HCV: hepatitis C virus; IDU, injection drug use; IQR, interquartile range; PWUD: people who use drugs.

^a^ 80 of 685 participants who tested positive for anti-HCV antibodies declined HCV-RNA testing and were excluded from the analysis.

^b^ Daily alcohol consumption > 50 g/day.

Values are expressed as numbers (percentage) and median (interquartile range). Analysis was performed using the Mann–Whitney U test and chi-square test. Statistically significant differences are shown in bold.

### Factors associated with active HCV infection among PWUD

**Figure 2:**
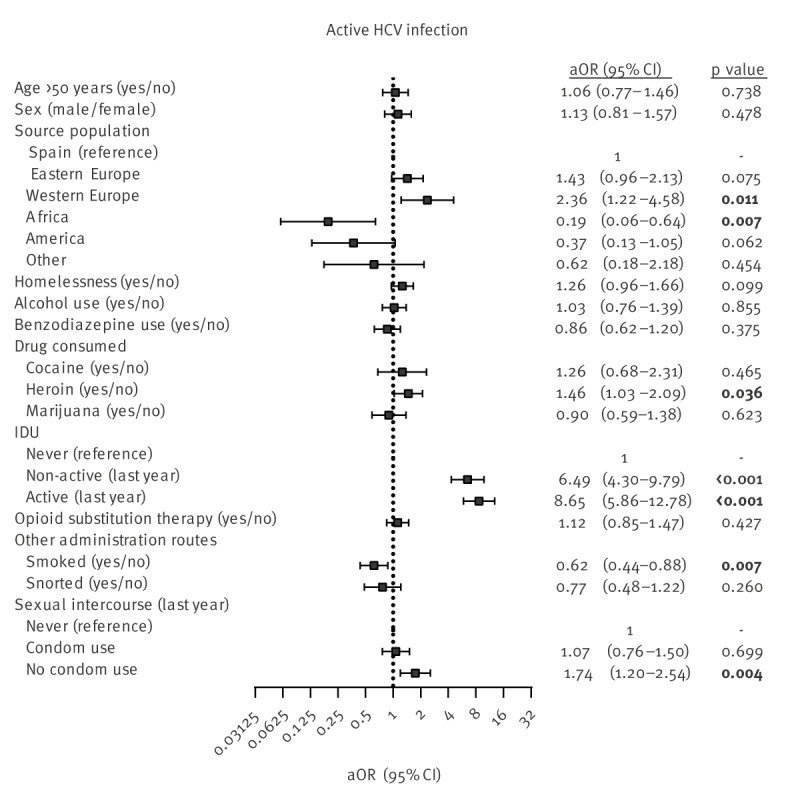
Risk factors for active HCV infection among PWUD, Madrid, Spain, 1 June 2017–3 April 2023 (n = 2,414 PCR tests)

The patient characteristics associated with HCV infection are shown in Supplementary Table S1. According to multivariate logistic regression analysis (Figure 2), factors directly associated with active HCV infection were Western Europe origin (aOR = 2.36; p = 0.011), heroin use (aOR = 1.46; p = 0.036), IDU non-active in the last year (aOR = 6.49; p < 0.001), IDU active in the past year (aOR = 8.65; p < 0.001) and no condom use in the previous year (aOR = 1.74; p = 0.004). On the other hand, factors inversely associated were African origin (aOR = 0.19; p = 0.007) and consumption of smoked drugs (aOR = 0.62; p = 0.007). These significant variables also manage to discriminate active HCV infection among PWUD with enough accuracy (AUC = 0.809), particularly the IDU alone (AUC = 0.771).

aOR: adjusted odds ratio; CI: confidence interval; HCV: hepatitis C virus; IDU: injection drug use; PWUD: people who use drugs.

Association analysis was performed using multivariable binary logistic regression. The reference category for dichotomous variables was always the one indicated second, i.e. female or no. Statistically significant differences (p < 0.05) are shown in bold.

### Temporal trend of HCV prevalence

**Figure 3:**
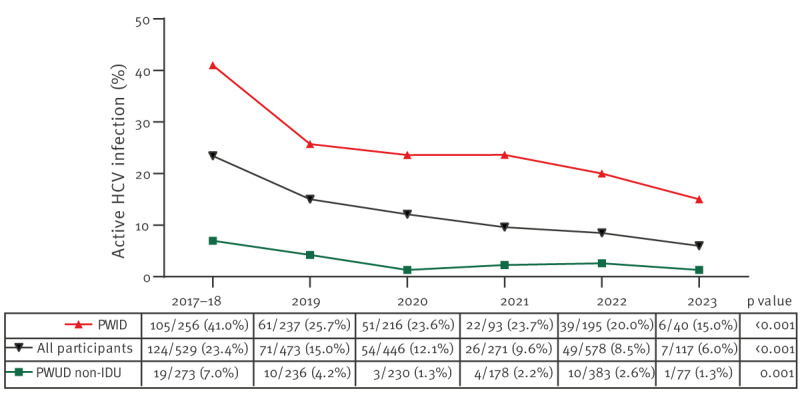
Prevalence of active HCV infection in the PWUD population, stratified by injecting drug use, Madrid, Spain, 1 June 2017–3 April 2023 (n = 2,414 PCR tests)

HCV: hepatitis C virus; IDU: injecting drug use; PWID: people who inject drugs; PWUD: people who use drugs.

Figure 3 shows the crude prevalence of active HCV infection in the entire population (PWUD) and the population stratified by IDU during the study period (2017–23). It is worth noting that 2017 and 2018 were combined because of a low participant count in 2018. The prevalence of active HCV infection exhibited a significant downward trend in the entire population (23.4% in 2017–18 to 6% in 2023, p < 0.001), among PWID (41% to 15%, p < 0.001), and PWUD non-IDU (7% to 1.3%, p < 0.001) (Figure 3). These trends were further evaluated by multivariate logistic regression, confirming that the prevalence of active HCV infection maintained a significant downward trend in the adjusted model for the entire population (aOR = 0.78; p < 0.001), PWID (aOR = 0.78; p < 0.001, and PWUD non-IDU (aOR = 0.78; p = 0.015).

Temporal trends of active HCV infection rates by calendar years were performed using logistic regression. Statistically significant differences are shown in bold.

The prevalence of IDU (active and inactive) and consumption of heroin and smoked drugs in PWUD during the study period (2017–23) is provided in Supplementary Figure S1. We found a significant decrease in the IDU and consumption of heroin rates (p < 0.001) and a slight increase in consumption of smoked drugs (p = 0.063) from 2017 to 2023. 

### Factors associated with HCV treatment initiation among PWUD

We also explored the factors associated with the initiation of HCV treatment among PWUD screened with active HCV infection (Supplementary Table S2), finding that PWUD who were homeless (aOR = 2.41; p = 0.003) and were on OST (aOR = 1.99; p = 0.016) had a higher likelihood of starting HCV treatment; while PWUD who had sexual intercourse without condom use in the previous year (aOR = 0.45; p = 0.041) had a lower likelihood of starting HCV treatment.

## Discussion

In this retrospective study, we assessed the risk factors and prevalence of active HCV infection among PWUD using a mobile screening unit serving hotspots in Madrid. Our main findings were: (i) among the risk factors for active HCV infection, IDU was the most prominent; (ii) active HCV infection among PWID during the study period (2017–23) was high, higher than in PWUD non-IDU; and (iii) the prevalence of active HCV infection decreased significantly from 2017 to 2023 across the entire population, as well as when stratified by IDU.

The WHO has set ambitious targets to eliminate HCV as a public health threat by 2030. However, some models suggest that only 24% of high-income countries are on track to achieve this goal, considering their current indicators for diagnosis and treatment [8]. While some studies have reported a global decrease in HCV cases from 2015 to 2020, ca 56.8 million viraemic infections were still estimated as of 1 January 2020, with only 22.7% (12.9 million) being diagnosed [3].

Our study identified that IDU (inactive and active in the last year) was the most relevant risk factor for active HCV infection among PWUD. This was evidenced by the high aOR and AUC values found for PWID and because heroin use was another risk factor for active HCV infection. These findings are consistent with those reported in the literature, as HCV transmission typically requires direct percutaneous exposure to the blood via blood transfusions, iatrogenic transmission or IDU [6], and people who use heroin often engage in higher-risk practices [25]. Although the burden of active HCV infection among PWID has decreased in Spain [15,16,19], the levels of active HCV infection found in our study point out the importance of prevention activities and harm-reduction strategies, such as needle and syringe programmes and OST, harm-reduction interventions, and HCV treatment [22,23]. Additionally, a cornerstone of harm-reduction strategies is encouraging people who use intravenous drugs to transition to less hazardous consumption methods, such as smoking using clean pipes. Such shifts could significantly lower the risk of infectious disease transmission, including hepatitis C, and improve overall well-being [26]. 

Age was identified as another risk factor, which could be explained by the fact that PWUD were primarily infected with HCV either through blood transfusions (before the systematic HCV screening implementation in the 1990s in Spain) or because of injecting drug use during the epidemic of the 1980s and 1990s [27].

Most studies examining active HCV infection trends in high-risk populations have primarily focused on PWID. For instance, Valerio et al. conducted an observational cohort study on PWID attending drug treatment clinics and needle and syringe programmes in Australia [28]. They showed a decrease in active HCV infection from 24% to 17% between 2018–19 and 2019–21, alongside an increase in HCV treatment from 66% to 74% during the same period. In the European context, a study performed on PWID at the needle and syringe programme in Uppsala, Sweden [29], reported a declining trend in HCV prevalence from 68.5% in 2013–16 to 20.9% in 2021, along with an increase in HCV treatment uptake and treatment outcome. In Spain, Fanciulli et al. assessed the epidemiological trends of HCV antibodies and active HCV infection in people living with HIV (PLWH) between 2015 and 2019 [30]. They found a decrease in HCV seroprevalence from 37.7% in 2015 to 28.6% in 2019, and active HCV infection from 22.1% in 2015 to 2.2% in 2019, with HCV treatment uptake increasing from 53.9% in 2015 to 95.0% in 2019 [30]. However, it should be noted that some authors have reported an increase in the incidence of hepatitis C in PLWH in the context of sexualised drug use (including slamsex and other chemsex practices) [31,32].

Our study found a prevalence of active HCV infection of 6.0% in the entire population of PWUD and 15.0% in PWID in 2023, which supposes a significant reduction from the initial prevalence recorded in 2017–18, which was 23.4% and 41.0%, respectively. However, it should be noted that despite these significant reductions, the HCV prevalence of active HCV infection in PWUD and PWID remains nearly 30 and 70 times higher, respectively, than the estimated prevalence of active HCV infection in the general population in Spain (0.2%) [4]. Consequently, efforts are still needed to reduce the burden of HCV infection in these high-risk groups.

The reduction in the prevalence of active HCV infection observed in our study, especially in PWID, could be attributed to several factors. These include improvements in diagnosis and linkage to care facilitated by decentralised, non-hospital-based approaches, simplified one-step diagnosis, and removal of treatment restrictions, which have helped overcome barriers faced by PWID. In particular, a single-visit PoC-based strategy for HCV screening using a mobile unit has proven to be highly effective, easy to implement, and cost-effective [18]. Additionally, having trained support workers using a mobile unit to help diagnose and prevent HCV infection is essential to reaching vulnerable people [10]. Moreover, robust public health campaigns have raised awareness about the risks associated with intravenous drug use. In this sense, our study found a significant downward trend in the IDU rate throughout the study period (2017–23), consistent with previous reports showing a decline in Spain's IDU rate in recent years [33]. This decrease in the IDU rate could have influenced a reduction in spread of active HCV infection in our study population as a result of the depletion of high-risk IDUs among those at risk of contracting hepatitis C [34]. Additionally, our study showed that the use of a mobile screening unit allowed us to achieve an HCV treatment initiation rate of almost 70% among PWUD, which is in line with other similar published studies [10,18]. In this sense, these strategies allow not only a rapid PoC diagnosis in a single visit but are also associated with a high linkage to care and initiation of HCV treatment. Our data also showed that being homeless and receiving OST were directly associated with starting HCV treatment, while sexual intercourse without condom use in the previous year was inversely associated with starting HCV treatment. Point-of-care testing linked to homeless-focused programmes has shown that homeless-experienced people had high rates of HCV testing, diagnosis, and linkage to care [35]. It is also known that integrating HCV screening into OST programmes facilitates patient access to HCV treatment, increasing rates of treatment completion and sustained virological responses [36]. Unprotected sexual intercourse is also a risk factor for active HCV infection, which indicates PWUD with sexual risk behaviour is a group of subjects of special interest for HCV elimination.

Finally, increasing evidence is showing that scaling up DAA therapy to reduce HCV incidence (treatment as prevention, TasP) could be cost-effective [37]. Two studies of PWID in Scotland [38] and England [39] concluded that the TasP strategy reduced the prevalence of hepatitis C. In addition, van Santen et al. [40] conducted a multinational study among PLWH, concluding that broad DAA access also has a TasP effect on decreasing HCV incidence in this population. In this regard, currently, hepatitis C treatment is free of charge for the entire population in Spain, although the prescription and administration of DAAs are exclusively hospital-based. As a result, PoC-based testing and other micro-elimination strategies have to face the additional challenge of linkage to care in hospital settings, which can limit acceptance rates and treatment adherence, especially among marginalised populations [18]. The prescription restrictions for DAA therapy should be removed to overcome this situation, allowing these treatments to be administered in healthcare and drug treatment centres [41]. Thus, decentralising HCV treatments would allow physicians to provide HCV therapy directly at the diagnostic site (test-and-treat strategy) [42].

Our study has some limitations. Firstly, the study is subject to potential selection bias. Some PWUD might not have participated in our study because of the absence of a financial incentive. Conversely, some high-risk groups, such as active PWUD, might have been more inclined to participate in order to receive a diagnosis. Secondly, risk behaviour data may contain biases or be incomplete, although it should be noted that the data were collected by trained personnel, which would have minimised errors. Thus, the frequency and route of drug use could be subject to recall bias, underestimating some associations. Finally, our study was performed on PWUD in a high-income country, which could limit the generalisability of our findings to other settings.

A large sample size was available to ensure greater stability, precision and representativeness of the results, which was a strength of the study. Additionally, the mobile screening unit facilitated the search for participants at critical meeting points for PWUD and screening for hepatitis C with methods adapted to population screening.

## Conclusion

Our study demonstrates a significant reduction in the prevalence of active HCV infection among PWUD, particularly in the primary risk group of PWID, which suggests that efforts in the prevention and treatment of HCV in Madrid, Spain, have had an impact on the control of HCV infection. 

## Supplementary Data

287000 Supplement
