# Supplementary material for: Decrease in active hepatitis C infection among people who use drugs in Madrid, Spain, 2017 to 2023: a retrospective study
Source: Euro Surveill. 2024 Jul 18;29(29):2300712. doi: 10.2807/1560-7917.ES.2024.29.29.2300712 (PMC11258947; doi:10.2807/1560-7917.ES.2024.29.29.2300712)
Supplement: Supplement [file 23-00712_RESINO_Supplement.pdf]

Eurosurveillance hosts this supplementary material as supporting information alongside the article [Decrease in active hepatitis C among people who use drugs in Madrid, Spain, 2017 to 2023: a retrospective study] on behalf of the authors, who remain responsible for the accuracy and appropriateness of the content. The same standards for ethics, copyright, attributions, and permissions apply to the article. Eurosurveillance does not edit supplements, and the journal is not responsible for the maintenance of any links or email addresses provided therein.

Supplementary materials

Supplementary Figure S1. Prevalence of IDU (active and inactive) and consumption of heroin and smoked drugs in PWUD during the study period, Madrid, Spain, 1 June 2017–3 April 2023 (n = 2,414 PCR tests). **Statistical analysis**: temporal trend rates by calendar years were calculated using logistic regression adjusted by patient characteristics. **Abbreviations**: PWUD, people who use drugs; IDU, injection drug use.

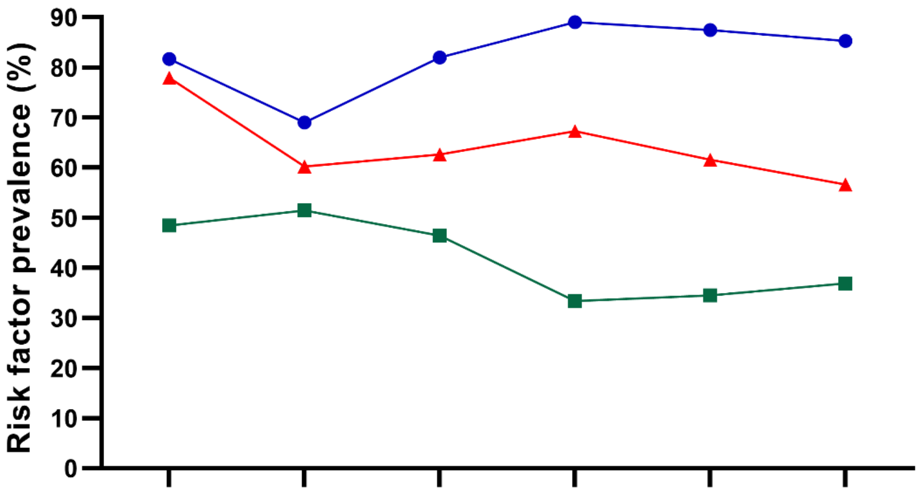

	2017-18	2019	2020	2021	2022	2023	p-value
IDU	256 (48.4%)	252 (51.4%)	179 (46.4%)	79 (33.3%)	179 (34.5%)	38 (36.9%)	**<0.001**
Heroin	412 (77.9%)	295 (60.2%)	288 (62.6%)	195 (67.2%)	386 (61.6%)	73 (56.6%)	**<0.001**
Smoked	432 (81.7%)	338 (69.0%)	377 (82.0%)	258 (89.0%)	548 (87.4%)	110 (85.3%)	0.063

Supplementary Table S1. Univariate analysis of risk factors for active HCV infection among PWUD, Madrid, Spain, 1 June 2017–3 April 2023 (n = 2,414 PCR tests).

	OR (95%CI)	p-value
**Age over 50 years (yes/no)**	0.97 (0.72 – 1.29)	0.813
**Sex (male/female)**	1.29 (0.97 – 1.72)	0.077
**Source population**		
**Spain (reference)**	1	-
**Eastern Europe**	2.16 (1.57 – 2.98)	**<0.001**
**Western Europe**	2.16 (1.20 – 3.87)	**0.010**
**Africa**	0.12 (0.04 – 0.39)	**<0.001**
**America**	0.17 (0.06 – 0.46)	**0.001**
**Other**	0.36 (0.11 – 1.18)	0.092
**Homeless (yes/no)**	1.33 (1.05 – 1.68)	**0.018**
**Alcohol intake (yes/no)**	0.70 (0.54 – 0.90)	**0.005**
**Benzodiazepine use (yes/no)**	0.94 (0.71 – 1.24)	0.641
**Drug consumed**		
**Cocaine (yes/no)**	1.87 (1.15 – 3.06)	**0.011**
**Heroin (yes/no)**	3.07 (2.28 – 4.15)	**<0.001**
**Marijuana (yes/no)**	0.43 (0.30 – 0.61)	**<0.001**
**IDU**		
**Never (reference)**	1	-
**Not active (over a year ago)**	7.87 (5.47 – 11.34)	**<0.001**
**Active (less than a year ago)**	13.28 (9.45 – 18.65)	**<0.001**
**Opioid substitution therapy (yes/no)**	2.10 (1.66 – 2.66)	**<0.001**
**Other administration routes**		
**Smoked (yes/no)**	0.55 (0.42 – 0.72)	**<0.001**
**Snorted (yes/no)**	0.31 (0.21 – 0.46)	**<0.001**
**Sexual intercourse (last year)**		
**Never (reference)**	1	-
**Condom use**	1.04 (0.78 – 1.38)	0.789
**No condom use**	1.35 (0.98 – 1.86)	0.065

Statistical analysis: Association analysis was performed using multivariable binary logistic regression. The reference category for dichotomous variables was always the one indicated in second place in brackets (female or no). Statistically significant differences are shown in bold. (*), Since we found only ten participants with HCV active infection from Africa, America, or other origins, these three categories were grouped for multivariate analysis. **Abbreviations**: aOR, adjusted odds ratio; HCV, hepatitis C virus; IDU, injection drug use; PWUD, people who use drugs.

Supplementary Table S2. Factors associated with the initiation of HCV treatment among PWUD screened with active HCV infection Madrid, Spain, 1 June 2017–3 April 2023 (n = 314).

	**aOR (95%CI)**	**p-value**
**Age over 50 years (yes/no)**	0.69 (0.34 - 1.40)	0.300
**Sex (male/female)**	0.80 (0.38 - 1.65)	0.544
**Source population**		
**Spain (reference)**	1	-
**Eastern Europe**	0.52 (0.25 - 1.11)	0.090
**Western Europe**	2.94 (0.61 - 14.27)	0.180
**Other ***	1.02 (0.22 - 4.68)	0.975
**Homeless (yes/no)**	2.41 (1.35 - 4.27)	**0.003**
**Alcohol intake (yes/no)**	1.43 (0.74 - 2.77)	0.284
**Benzodiazepine use (yes/no)**	1.12 (0.53 - 2.36)	0.764
**Drug consumed**		
**Cocaine (yes/no)**	1.33 (0.40 - 4.41)	0.637
**Heroin (yes/no)**	0.94 (0.44 - 2.01)	0.875
**Marijuana (yes/no)**	0.75 (0.33 - 1.74)	0.508
**IDU**		
**Never (reference)**	1	-
**Not active (over a year ago)**	2.36 (0.97 - 5.71)	0.057
**Active (less than a year ago)**	1.28 (0.56 - 2.94)	0.562
**Opioid substitution therapy (yes/no)**	1.99 (1.14 - 3.46)	**0.016**
**Other administration routes**		
**Smoked (yes/no)**	0.98 (0.48 - 1.98)	0.944
**Snorted (yes/no)**	1.25 (0.47 - 3.32)	0.649
**Sexual intercourse (last year)**		
**Never (reference)**	1	
**Condom use**	0.67 (0.31 - 1.41)	0.287
**No condom use**	0.45 (0.21 - 0.97)	**0.041**

Statistical analysis: Association analysis was performed using multivariable binary logistic regression. The reference category for dichotomous variables was always the one indicated in second place in brackets (female or no). Statistically significant differences are shown in bold. (*), Since we found only ten participants with HCV active infection from Africa, America, or other origins, these three categories were grouped for multivariate analysis. **Abbreviations**: aOR, adjusted odds ratio; HCV, hepatitis C virus; IDU, injection drug use; PWUD, people who use drugs.
